# Supplementary material for: Risk Factors Predisposing to Angina in Patients with Non-Obstructive Coronary Arteries: A Retrospective Analysis
Source: J Pers Med. 2022 Jun 27;12(7):1049. doi: 10.3390/jpm12071049 (PMC9318432; doi:10.3390/jpm12071049)
Supplement: Supplementary file 1 [file jpm-12-01049-s001.zip › jpm-1739362-supplementary.pdf]

Journal of Personalized Medicine

MDPI

Supplementary material

Table S1. Univariable logistic regression analysis—numerical values.

Parameter	OR	95% CI	p-value
Age	0.979	0.958 – 1.001	p=0.058
BMI	0.996	0.949 – 1.044	p=0.861
Female sex**	2.00	1.201 – 3.332	p=0.008
Living on the countryside	0.634	0.375 – 1.072	p=0.089
Arterial hypertension***	0.178	0.089 – 0.354	p<0.001
Diabetes mellitus (all types)*	0.548	0.321 – 0.938	p=0.028
Diabetes mellitus type 2	0.584	0.339 – 1.005	p=0.052
Heart failure	1.020	0.604 – 1.721	p=0.941
Family history of CVD	1.119	0.654 – 1.914	p=0.681
TC*	1.239	1.023 – 1.500	p=0.028
LDL-C**	1.436	1.134 – 1.818	p=0.003
HDL-C	1.161	0.654 – 2.059	p=0.611
non-HDL-C*	1.250	1.021 – 1.531	p=0.031
LDL-C/HDL-C**	1.513	1.132 – 2.022	p=0.005
non-HDL-C/HDL-C*	1.241	1.001 – 1.538	p=0.049
TG	0.919	0.701 – 1.205	p=0.541
cTnI	1.032	0.918 – 1.160	p=0.603
hs-CRP	0.992	0.974 – 1.010	p=0.357
EF	1.024	0.999 – 1.049	p=0.065
LVEDD	0.985	0.955 – 1.016	p=0.337

*p<0.05; **p<0.01; ***p<0.001

BMI—body mass index; cTnI—cardiac troponin I; CVD—cardiovascular diseases; EF—ejection fraction; HDL-C—high-density lipoprotein cholesterol; hs-CRP—high-sensitive C-reactive protein; LDL-C—low-density lipoprotein cholesterol; LVEDD—left ventricle end-diastolic diameter; non-HDL-C—non-high-density lipoprotein cholesterol; TC—total cholesterol; TG—triglycerides.

An additional analysis regarding only patients with hemodynamically significant lesions in one or multiple coronary arteries has been performed. The results of the analysis are presented in Tables S2-S5.

We found that the median BMI was significantly higher in the group with only one obstructed coronary artery and the multivariable regression analysis revealed that BMI could be recognized as an independent risk factor, decreasing the risk for multiple lesions (OR=0.908, 95%CI: 0.832 – 0.990; p=0.030). This unexpected result might fit into the pattern that the lower severity of CAD, the more underestimation from the patients and doctors it has, same as we presented in the case of the lipid profile in our work. Furthermore, the past myocardial infarction or past PCI were unsurprisingly reported more frequently in the multiple obstructed coronary arteries group and were both considered the independent risk factors (OR=2.433 and OR=3.084, respectively). Notably, we observed that the use of mineralocorticoid receptor antagonists (MRA) was more common in the group with multiple coronary arteries obstructed, albeit there were no statistically significant differences in the prevalence of heart failure or hypertension. Interestingly, statin therapy did not differ between the groups.

Table S2. Additional OCAD group analysis: population characteristics.
Group 1—only one coronary artery obstructed; group 2—multiple coronary arteries obstructed.

Parameter	Group 1 *N (% value) or median (q1–q3)*	Group 2 *N (% value) or median (q1–q3)*	p-value
Number of participants	72 (100%)	56 (100%)	
Personal characteristics			
Gender (females)	24 (33%)	14 (25%)	p=0.368
Living in the countryside/in the city	22/50 (31%/69%)	23/33 (41%/59%)	p=0.188
Age	68.00 (59.00–75.00)	69.00 (63.00–75.00)	p=0.334
BMI (kg/m^2)**	29.38 (26.32–33.33)	27.07 (24.46–30.48)	p=0.008
Comorbidities			
Arterial hypertension	66 (92%)	51 (91%)	p=0.919
Diabetes mellitus (all types)	25 (35%)	21 (38%)	p=0.652
DM2	23 (32%)	20 (36%)	p=0.562
Heart failure	20 (28%)	18 (32%)	p=0.497
Past myocardial infarction**	18 (25%)	29 (52%)	p=0.002
Past PCI (at least one)***	25 (35%)	38 (68%)	p<0.001
Family history of CVD	36 (56%)<br>Total N: 64 (100%) [a]	26 (59%)<br>Total N: 44 (100%) [a]	p=0.769
Addictions			
Alcohol consumption	Total N: 52 (100%) [a]	Total N: 32 (100%) [a]	
No	44 (85%)	26 (81%)	p=0.610
Occasionally	7 (13%)	6 (19%)	
Frequently	1 (2%)	0 (0%)	
Smoking	Total N: 66 (100%) [a]	Total N: 44 (100%) [a]	
Never	33 (50%)	21 (48%)	p=0.749
Active smoker	14 (21%)	8 (18%)	
Smoking in the past	19 (29%)	15 (34%)	

*p<0.05; **p<0.01; ***p<0.001
[a] In the case of family history of CVD, alcohol consumption, and smoking, the total N differs from the number of participants in each group due to the missing information in medical records.

BMI—body mass index; CVD—cardiovascular disease; DM2—type 2 diabetes mellitus; OCAD—obstructive coronary artery disease; PCI—percutaneous coronary intervention.

Table S3. Additional OCAD group analysis: laboratory and echocardiographic parameters.
Group 1—only one coronary artery obstructed; group 2—multiple coronary arteries obstructed.

Parameter	Unit	Group 1 *median (q1–q3)*	Group 2 *median (q1–q3)*	p-value
Lipid parameters				
TC	mmol/L	4.06 (3.40–5.03)	4.35 (3.59–4.98)	p=0.545
LDL-C	mmol/L	2.10 (1.50–2.80)	2.10 (1.60–2.60)	p=0.958
HDL-C	mmol/L	1.44 (1.21–1.57)	1.31 (1.06–1.68)	p=0.180
Non-HDL-C	mmol/L	2.62 (2.06–3.27)	2.95 (2.30–3.53)	p=0.407
TG	mmol/L	1.14 (0.76–1.52)	1.26 (0.85–1.80)	p=0.264
LDL-C/HDL-C ratio	1	1.49 (1.09–1.88)	1.55 (1.23–2.13)	p=0.373
Non-HDL-C/HDL-C ratio	1	1.91 (1.42–2.46)	2.09 (1.46–2.73)	p=0.128
White blood parameters and C-reactive protein				
White blood count	10^9/L	7.31 (6.32–9.11)	6.76 (5.41–8.29)	p=0.126

Neutrophil count	10^9/L	5.00 (3.97–6.14)	4.19 (3.20–6.08)	p=0.208
Eosinophil count	10^9/L	0.13 (0.08–0.21)	0.12 (0.07–0.21)	p=0.513
Basophil count	10^9/L	0.03 (0.02–0.04)	0.03 (0.02–0.04)	p=0.254
Lymphocyte count	10^9/L	1.76 (1.29–1.96)	1.48 (1.19–1.79)	p=0.089
Monocyte count	10^9/L	0.44 (0.37–0.54)	0.41 (0.32–0.49)	p=0.188
NLR	1	2.73 (2.26–3.89)	3.06 (2.17–4.47)	p=0.778
LMR	1	3.72 (2.90–4.59)	3.73 (2.54–4.57)	p=0.800
hs-CRP	mg/L	4.90 (3.20–7.90)	5.40 (1.80–6.90)	p=0.307
Troponins				
cTnI	ng/mL	0.000 (0.000–0.011)	0.002 (0.000–0.018)	p=0.456
Echocardiographic parameters				
EF	%	55.00 (50.00–60.00)	55.00 (47.50–60.00)	p=0.875
LA diameter	mm	43.00 (40.00–46.00)	42.00 (37.50–48.00)	p=0.723
LVEDD	mm	51.00 (47.00–55.00)	53.00 (47.50–57.00)	p=0.390
RVD	mm	32.00 (30.00–35.00)	33.00 (30.00–35.00)	p=0.936
IVS thickness	mm	11.00 (10.00–12.00)	11.00 (10.00–13.00)	p=0.645
PW thickness	mm	11.00 (10.00–12.00)	11.00 (10.00–12.00)	p=0.881

*p<0.05; **p<0.01; ***p<0.001

cTnI—cardiac troponin I; EF—ejection fraction; HDL-C—high-density lipoprotein cholesterol; hs-CRP—high-sensitive C-reactive protein; IVS—interventricular septum; LA—left atrium; LDL-C—low-density lipoprotein cholesterol; LMR—lymphocyte/monocyte ratio; LVEDD—left ventricle end-diastolic diameter; NLR—neutrophil/lymphocyte ratio; non-HDL-C—non-high-density lipoprotein cholesterol; OCAD—obstructive coronary artery disease; PW—posterior wall; RVD—right ventricle diameter; TC—total cholesterol; TG—triglycerides.

Table S4. Additional OCAD group analysis: univariable and multivariable logistic regression. Group 1—only one coronary artery obstructed; group 2—multiple coronary arteries obstructed (modelled class).

**Univariable logistic regression analysis**			
**Parameter**	**OR**	**95% CI**	**p-value**
Age	1.017	0.981-1.054	p=0.361
BMI**	0.896	0.825-0.974	p=0.009
Female sex	0.698	0.318-1.530	p=0.369
Living on the countryside	0.609	0.290-1.278	p=0.190
Arterial hypertension	0.938	0.270-3.250	p=0.919
Diabetes mellitus (all types)	1.184	0.568-2.468	p=0.653
Diabetes mellitus type 2	1.247	0.591-2.629	p=0.562
Heart failure	1.306	0.604-2.824	p=0.498
Past myocardial infarction**	3.233	1.512-6.915	p=0.003
Past PCI (at least one)***	3.940	1.863-8.331	p<0.001
Family history of CVD	1.123	0.516-2.446	p=0.769
TC	0.969	0.726-1.293	p=0.831
LDL-C	0.891	0.616-1.289	p=0.540
HDL-C	0.507	0.201-1.280	p=0.150
non-HDL-C	1.048	0.764-1.437	p=0.772
LDL-C/HDL-C	1.294	0.786-2.129	p=0.311
non-HDL-C/HDL-C	1.431	0.979-2.092	p=0.064
TG	1.462	0.969-2.206	p=0.070
cTnI	0.015	0.000-999.9	p=0.603
hs-CRP	0.818	0.529-1.263	p=0.364
EF	0.996	0.957-1.036	p=0.834
LVEDD	1.013	0.961-1.069	p=0.627
**Multivariable logistic regression analysis**[a]			
**Parameter**	**OR**	**95% CI**	**p-value**
Model 1			
BMI*	0.908	0.832 – 0.990	p=0.030
Past PCI (at least one)**	3.084	1.421 – 6.693	p=0.004
Model 2			
BMI*	0.909	0.835 – 0.991	p=0.030
Past myocardial infarction*	2.433	1.105 – 5.359	p=0.027

*p<0.05; **p<0.01; ***p<0.001

[a] Because past PCI and past myocardial infarction appeared collinear, two different regression models were created.

BMI—body mass index; cTnI—cardiac troponin I; CVD—cardiovascular diseases; EF—ejection fraction; HDL-C—high-density lipoprotein cholesterol; hs-CRP—high-sensitive C-reactive protein; LDL-C—low-density lipoprotein cholesterol; LVEDD—left ventricle end-diastolic diameter; non-HDL-C—non-high-density lipoprotein cholesterol; PCI—percutaneous coronary intervention; TC—total cholesterol; TG—triglycerides.

Table S5. Additional OCAD group analysis: summary of pre-hospital medications.
Group 1—only one coronary artery obstructed; group 2—multiple coronary arteries obstructed.

Medicine	**Number of participants** *N (% value)* **Total N: 125** [a]		p-value
	Group 1 N: 70 (100%)	Group 2 N: 55 (100%)	
ACE-I / ARB	60 (85.71%)	42 (76.36%)	p=0.180
Acetylsalicylic Acid	46 (65.71%)	34 (61.82%)	p=0.652
b-Blockers	48 (68.57%)	44 (80.00%)	p=0.150
Ca-blockers	23 (32.86%)	22 (40.00%)	p=0.409
Diuretics (thiazides + loop diuretics)	37 (52.86%)	26 (47.27%)	p=0.535
Metformin	16 (22.86%)	16 (29.09%)	p=0.428
MRA*	10 (14.29%)	18 (32.73%)	p=0.014
Nitrates	6 (8.57%)	5 (9.09%)	p=0.919
Statins	58 (82.86%)	46 (83.64%)	p=0.908
Approximate atorvastatin dose[b]	40 (20–60)	40 (20–40)	p=0.118
>20 mg/d	27 (38.57%)	29 (52.73%)	p=0.114
≤20 mg/d	43 (61.43%)	26 (47.27%)	p=0.114
Trimetazidine	11 (15.71%)	6 (10.91%)	p=0.437
NOAC	7 (10.00%)	6 (10.91%)	p=0.869
VKA	5 (7.14%)	2 (3.64%)	p=0.694

*p<0.05; **p<0.01; ***p<0.001
[a] The total N differs from the number of participants in each group due to the missing information in medical records.
[b] Calculation based on [30].

ACE-I—angiotensin-converting enzyme inhibitors; ARB—angiotensin II receptor blockers; MRA—mineralocorticoid receptor antagonists; NOAC—novel oral anticoagulants; OCAD—obstructive coronary artery disease; VKA—vitamin K antagonists.
